# Cutaneous Leishmaniasis in dogs: is high seroprevalence indicative of a reservoir role?

**DOI:** 10.1017/S0031182015000475

**Published:** 2015-05-20

**Authors:** JOSÉ E. CALZADA, AZAEL SALDAÑA, KADIR GONZÁLEZ, CHYSTRIE RIGG, VANESSA PINEDA, ANA MARÍA SANTAMARÍA, INDRA RODRÍGUEZ, NICOLE L. GOTTDENKER, MARCIA D. LAURENTI, LUIS F. CHAVES

**Affiliations:** 1 Departamento de Parasitología, Instituto Conmemorativo Gorgas de Estudios de Salud (ICGES), Ciudad de Panamá, República de Panamá; 2 Facultad de Medicina Veterinaria, Universidad de Panamá, Ciudad de Panamá, República de Panamá; 3 Department of Veterinary Pathology, College of Veterinary Medicine, University of Georgia, Athens, Georgia, USA; 4 Departamento de Patología, Facultade de Medicina, Universidade de São Paulo, São Paulo, Brasil; 5 Institute of Tropical Medicine (NEKKEN), Nagasaki University, 852-8523 Sakamoto 1-12-4, Nagasaki, Japan; 6 Programa de Investigación en Enfermedades Tropicales (PIET), Escuela de Medicina Veterinaria, Universidad Nacional, Heredia, Costa Rica

**Keywords:** *Canis familiaris*, *Leishmania panamensis*, reservoirs, endemicity, force of infection, catalytic models

## Abstract

American cutaneous leishmaniasis (ACL) is a complex disease with a rich diversity of animal host species. This diversity imposes a challenge, since understanding ACL transmission requires the adequate identification of reservoir hosts, those species able to be a source of additional infections. In this study we present results from an ACL cross-sectional serological survey of 51 dogs (*Canis familiaris*), where we used diagnostic tests that measure dog's exposure to *Leishmania* spp. parasites. We did our research in Panamá, at a village that has undergone significant ecosystem level transformations. We found an ACL seroprevalence of 47% among dogs, and their exposure was positively associated with dog age and abundance of sand fly vectors in the houses of dog owners. Using mathematical models, which were fitted to data on the proportion of positive tests as function of dog age, we estimated a basic reproductive number (*R*_0_ ± s.e.) of 1·22 ± 0·09 that indicates the disease is endemically established in the dogs. Nevertheless, this information by itself is insufficient to incriminate dogs as ACL reservoirs, given the inability to find parasites (or their DNA) in seropositive dogs and previously reported failures to experimentally infect vectors feeding on dogs with ACL parasites.

## INTRODUCTION

American cutaneous leishmaniasis (ACL) is a complex disease system, with several interacting socio-ecological factors underpinning its transmission dynamics (Chaves *et al.* 2008*a*). Along the social axis of its complexity, over the years, it has become clear that ACL is a disease intimately related with patterns of inequity in wealth distribution and poverty (Alvar *et al.* 2012). Along the ecological axis of its complexity, ACL is a disease system that serves as a model to study major ecological processes and biodiversity patterns, beginning with a rich diversity of vectors, animal host species (Christensen and de Vasquez, 1982; Christensen *et al.* 1983; Dutari and Loaiza, 2014) and parasites whose interactions change with ecosystem-wide disturbances (or irreversible shifts), such as deforestation and biodiversity destruction (Alexander *et al.* 2001; Quintana *et al.* 2010). When social and ecological axes converge, ACL emerges as a disease with a large diversity of epidemiological patterns, where, for example, poor housing quality may impair vector control success (Chaves *et al.* 2013), and the identification of major vertebrate reservoir hosts becomes a major challenge in settings where domestic animals and wildlife species co-exist (Chaves *et al.* 2007).

Reservoirs are mammal hosts with dynamic feedbacks in pathogen transmission, i.e. animals able to serve as source of pathogens to vectors and/or new hosts (Viana *et al.* 2014). In the case of ACL this equates to mammal species that are a common source of infection for sand flies, something possible when a given mammal species: (i) is commonly bitten by sand flies that also bite humans, (ii) has life history and immunological traits that allow *Leishmania* spp. parasites development and transmission after being infected, (iii) and where parasites can be easily isolated from blood or tissues where sand flies can get infected. Interestingly, ACL reservoir hosts do not necessarily present ACL associated pathologies and the infection does not need to be endemically established in their populations (Ashford, 1997; Chaves *et al.* 2007).

Reservoirs are particularly difficult to identify in ACL given the richness of recorded hosts for New World Cutaneous (NWC) *Leishmania* spp. parasites and the catholic blood feeding patterns of dominant vector species (Christensen *et al.* 1983; Dutari and Loaiza, 2014). Among ACL vertebrate host species, the domestic dog *Canis familiaris* has been the centre of controversy and there is a lack of consensus regarding its role as reservoir of NWC *Leishmania* spp. parasites (Falqueto *et al.* 1991; Reithinger and Davies, 1999; Dantas-Torres, 2007). From the initial report of dog infections in an endemic rural focus of ACL (Pedroso, 1913) and the suspicion of their role as likely reservoirs in the first urban ACL epidemic reported in the New World (Aragão, 1927), the high seroprevalence and the association between prevalence patterns in humans and dogs have been taken as major indicatives of a reservoir role for dogs in ACL epidemiology (Falqueto *et al.* 1991; Reithinger *et al.* 2003*b*). Nevertheless, studies considering *Leishmania* infections in other domestic animals have shown that equids, i.e. horses, donkeys and mules, were the likely reservoir in systems where dogs were originally suspected as NWC *Leishmania* reservoirs (Aguilar *et al.* 1989) a fact further reinforced by the low success probability of sand fly vectors at getting infected when biting dogs with NWC *Leishmania* spp. (Herrer and Christensen, 1976*b*; Hernández *et al.* 2006; Travi *et al.* 2006) and by mathematical modelling based on epidemiological reports where data on potential reservoir hosts including dogs and other species were collected (Chaves *et al.* 2007, 2008*b*).

In Panamá the main parasite currently causing ACL in humans is *Leishmania panamensis* (Chaves *et al.* 2014) and extensive research at the Gorgas Memorial Research Laboratory (current ICGES) in the 1960–1980s period showed the two toed sloth, *Choloepus hoffmani,* to be the main reservoir for *L. panamensis* (Christensen and Herrer, 1979; Herrer and Christensen, 1980). Furthermore, several studies suggested that dogs unlikely are ACL reservoirs in Panamá (Christensen and Herrer, 1973; Herrer and Christensen, 1976*a*, *b*). Similarly, to the best of our knowledge, the only other instance where ACL was studied in dogs with *L. panamensis* as the only circulating parasite was in the coast of Ecuador, and it did not find support for dogs as reservoirs (Dereure *et al.* 1994). Nevertheless, in Panamá, recent ACL epidemics in rural settings with a relatively recent history of major environmental change and urbanization (Saldaña *et al.* 2013) have raised questions about possible changes in the epidemiology of ACL. An outstanding question is whether dogs are becoming dominant reservoir hosts driving the dynamics of ACL transmission (Dantas-Torres, 2007). Here, we present results from a cross-sectional study in dogs from Trinidad de Las Minas, an endemic ACL rural community in Panamá Province, República de Panamá. We asked whether dogs were suitable reservoirs for ACL parasites, by attempting to detect ACL parasites and looking at their ACL seroprevalence patterns. We also studied the potential risk factors associated with ACL seropositive dogs. Briefly, we evaluated exposure to NWC *Leishmania* spp. in a population of 51 dogs by an indirect immunofluorescence assay test (IFAT) and an enzyme-linked immunosorbent assay (ELISA) using *L. panamensis* isolates as antigens. We also diagnosed active infections by parasite culture and polymerase chain reaction (PCR) tests from blood samples. Given the absence of a suitable gold standard (Reithinger *et al.* 2000), reflected in our study by the absence of CL lesions, negative PCR and parasite isolation results, as well as, the lack of agreement between the serological tests; we employed Bayesian methods to estimate the sensitivity and specificity of the serological tests (Branscum *et al.* 2005). We then evaluated risks factors for canine ACL seropositive reactions. Given that *R*_0_, the basic reproduction number, which is defined as the expected number of new infections in a susceptible host population following the introduction of an infected host (Anderson and May, 1991), has been reified as a criterion to infer that dogs are ACL reservoirs when larger than one, i.e. when *R*_0_ > 1 (Reithinger *et al.* 2003*a*), we employed maximum likelihood statistical methods to estimate the force of infection and *R*_0_ from an age specific seroprevalence curve, to better illustrate how this parameter should be interpreted.

## METHODS

### Study site

We studied canine cutaneous leishmaniasis exposure in dogs from Trinidad de Las Minas (8°46′32″ N; 79°59′45″ W), Panamá province, República de Panamá. Climate in the area is unimodal, with rainy (April–November) and dry (December–March) seasons. Rainfall ranges from 28 to 570 mm^3^ per month. Temperature is nearly constant with a year-round 26 °C average. Over recent years the native vegetation of our study site has been destroyed mainly for agricultural development, and the forest has become patchy and transitional (Chaves *et al.* 2013). We performed a census of dogs in May 2010, counting and recording individual data from all dogs in 24 households. We surveyed 24 houses; out of 198 in the village, because our resources were limited, especially the number of light traps available for Sand Fly (SF) sampling. Nevertheless, all houses enrolled in the study had confirmed SF presence (by the residents), had similar eco-epidemiological conditions and householders provided written informed consent to take samples from the animals. We also want to mention that assuming two dogs per house (Herrer and Christensen, 1976*a*, *b*), i.e. an approximate dog population size of 396 dogs in the whole village, a sample size of 48 dogs is large enough to detect a seroprevalence of at least 15% with a 10% precision, i.e. with 95% confidence intervals ranging from 5 to 25% (Sokal and Rohlf, 1994), prevalence values that have been previously recorded in Panamá (Herrer and Christensen, 1976*a*, *b*) and elsewhere canine cutaneous leishmaniasis is endemic in the New World (Reithinger and Davies, 1999).

### Dog sampling

Households from the studied area were individually visited. Every dog in each household was physically examined with emphasis on the dog's skin and mucosa. Blood samples were collected from the cephalic vein under minimal stress with the consent and in the presence of the owners. Age, sex and body condition of each dog were assessed during sample collection following standard procedures described by Fung *et al.* (2014). Dog age was calculated based on the owner information, and independently corroborated by dentition and tartar deposition patterns. The presence of ectoparasites (such as fleas and ticks) was visually assessed with the help of a hand-held magnifying lens. Blood samples were used for parasite isolation and for serological/molecular ACL diagnosis. Also, if skin lesions compatible with ACL were observed, scrapings were collected and spotted onto filter paper for molecular diagnosis. All dogs were dewormed after sampling.

### Parasite isolation and molecular analysis

Blood samples were inoculated into biphasic Senekjie's medium with M199 (Sigma, St Louis, MO, USA) to attempt parasite isolation. Culture tubes were incubated at 26 ⁰C and checked for *Leishmania* spp. promastigote presence every 7 days during 28 days. DNA was extracted from whole blood using the DNeasy extraction kit (Qiagen, Valencia, CA) according to the manufacturer's protocols. Primers targeting the entire 750-bp minicircle *of Leishmania Viannia* sp. (Vergel *et al.* 2005) and the heat-shock protein 70 of New World *Leishmania* (Garcia *et al.* 2004) were used for PCR diagnosis. Amplification reactions were performed in a final volume of 50 *μ*L. Internal positive and negative controls were used during each PCR analysis. Positive controls consisted of isolates from humans from the area (Miranda *et al.* 2012). A blood sample of a seronegative healthy dog from Ciudad de Panamá (non-endemic to *Leishmania*) was included in every PCR run as a negative extraction control. In every PCR we also included one reaction which contained only the reagents and no template, as a negative PCR control.

### ACL serology in dogs

The presence of IgG antibodies against *L. panamensis* was assessed by ELISA and IFAT. For the ELISA, a total crude antigen derived from *L. panamensis* promastigotes (MHOM/PA/98/WR/2306) was implemented following the exact same steps described by Lemos *et al.* (2008) and Colombo *et al.* (2011). The detection of antibodies to *L. panamensis* by IFAT was carried out using a suspension of promastigotes in buffered saline solution following the protocol described, in full detail, by the World Organization for Animal Health (OIE, 2008).

### Entomological, epidemiological and ecological variables at the household level

A series of variables, potentially associated with canine cutaneous leishmaniasis pathogen exposure, were quantified for each one of the households (and/or their peridomiciliary environments) enrolled in our study.

#### Entomological variables

SF were sampled once per month (April–June 2010) using modified light traps with an additional LED light (Chaves *et al.* 2013). Two traps were set from 6 pm to 6 am, at 2 m height in each household, and at the same site during the three sampling periods. One trap was placed inside the main room of the household (domicile) and the other trap over vegetation within a 50 m radius from the house (peridomicile). SF were removed from the traps and stored at −20 °C to kill the samples, which were subsequently preserved in 70% alcohol, separated by sex and identified to the species level based on the male genitalia and female spermathecae following the taxonomic key of Young and Duncan (1994). For the analysis, we employed the average from the three monthly observations, in order to have a reliable estimate of SF abundance. A detailed inventory of the collected sand flies species was presented by Calzada *et al.* (2013).

#### Epidemiological variables

We recorded the number of humans, the number of humans with active and parasitologically confirmed leishmaniasis lesions, the number of humans with past leishmaniasis lesions in each household. A full and detailed description of epidemiological data collection and patterns of clinical cutaneous leishmaniasis in humans at the study site was presented by Saldaña *et al.* (2013).

#### Ecological variables

For each household we estimated: a housing destituteness index, which quantified how different elements of housing construction and materials rendered houses differentially suitable habitats for SF; a peridomicile index, that quantified the abundance and availability of adult SF resting sites in a peridomicile; a vegetation index, that measured natural vegetation vertical structure; the richness, i.e. number of species, of domestic and wildlife animals; an index of domestic animal abundance, which weighted the abundance of different domestic species belonging to a household; and an index of wild animal presence, which weighted the commonness of different wildlife species sighted by householders. A detailed description of data collection and the estimation of each index was presented by Chaves *et al.* (2013).

### Ethical approval

This study was evaluated and approved by the National Review Board, Comité Nacional de Bioética de la Investigación, Instituto Conmemorativo Gorgas de Estudios de la Salud, Ciudad de Panamá, República de Panamá (561/CNBI/ICGES/06), and by ICGES Institutional Animal Care and Use Committee (IACUC, 2006/02). The study was in accordance with law No. 23 of January 15 1997 (Animal Welfare Assurance) of República de Panamá.

### IFAT seropositive assignation

The samples were considered positive when titers were ≥1:40 (Dantas-Torres *et al.* 2010). The positive control was a serum sample from a dog naturally infected with *L. Viannia panamensis* from central Panama with a 1:160 titer.

### ELISA seropositive assignation from the optical densities

We assigned as seropositive all dogs whose averaged optical density from the ELISA was above the mean plus three standard deviations (s.d.) of the distribution with the smallest mean (i.e. that of the likely seronegative individuals) in a finite mixture model, for details see Supplement S1. This method was chosen because of its robustness to small changes in antibody titres that can emerge from seasonality and/or small variations in laboratory assay performance (Stewart *et al.* 2009), problems already identified for canine leishmaniasis serodiagnosis (Dye *et al.* 1993). For the distribution with the smallest mean in the mixture we estimated a mean (±s.e.) of 0·102 ± 0·038, which leads to a seropositivity threshold of 0·216, i.e. any dog whose ELISA optical density was above this value could be considered positive, Fig. S1 shows the distribution of the optical densities from the ELISA test.

### Sensitivity and specificity for the IFAT and ELISA as diagnostics of canine cutaneous leishmaniasis caused by *Leishmania (Viannia) panamensis*

Sensitivity, the ability of a test to diagnose a true infection, and specificity, the ability of a test to avoid false negative diagnostics is generally assessed in the presence of a ‘gold standard’, for example, the direct observation of a parasite or its DNA amplification via PCR (Branscum *et al.* 2005). Nevertheless, canine cutaneous leishmaniasis is a system where a ‘gold standard’ is likely not plausible, given the limited tissues where positive PCRs could be expected, and observed differences in diagnosis for tissues from the same dog (Reithinger *et al.* 2000, 2002). In fact, we attempted parasite isolation and tested parasite presence in blood samples using PCR protocols described in (Miranda *et al.* 2012), all results were negative, probably because of low parasite loads (Padilla *et al.* 2002) and the low haematogenicity of *Leishmania* (*Viannia*) spp. in dogs (Dereure *et al.* 1994; Reithinger and Davies, 2002). Therefore, we employed the method developed by Dendukuri and Joseph (2001) to estimate the sensitivity and specificity of two serological diagnostic tests in one population, which employs a Bayesian framework for parameter inference, for details see Supplement S2. For the analysis we assumed the following priors: uniform distributions for the covariance of positive and negative tests, an uninformative beta distribution for the real prevalence in the population, beta distributions with mode = 0·90 and 5th percentile = 0·70 for the specificity and sensitivity of the ELISA, a beta distribution with mode = 0·70 and 5th percentile = 0·50 for the IFAT sensitivity, and a beta distribution with mode = 0·80 and 5th percentile = 0·60 for the IFAT specificity. These distributions were based on reported sensitivities for ELISA and IFAT tests for canine cutaneous leishmaniasis, and reflect the observation that ELISA tests tend to outperform IFAT tests in dogs (Supplement S3). Posterior inferences were based on 100 000 realizations that followed 10 000 burned-in realizations for the Markov Chain Monte Carlo (MCMC) stabilization. MCMC convergence was assessed by running multiple chains with dispersed starting values. Following the recommendations of Branscum *et al.* (2005), we also performed a parameter sensitivity analysis, described in Supplement S2. The analyses described in this section were implemented in the BUGS statistical software modifying the code developed by Branscum *et al.* (2005). Since our study area is also endemic for the etiologic agent of Chagas disease, *Trypanosoma cruzi*, we also assessed the possibility of cross-reaction for Chagas-Leishmania parasites (Padilla *et al.* 2002; Castro *et al.* 2007; Gil *et al.* 2011) by performing a *T. cruzi* adsorption of *Leishmania* spp. seropositive sera (Padilla *et al.* 2002) and by evaluating three different *T. cruzi* tests on samples from each individual dog. We evaluated how likely were the results similar by mere chance through the estimation of Cohen's coefficient of agreement Kappa (Cohen, 1960), between the *Leishmania* IFAT and ELISA diagnostic tests with a rapid immunochromatographic test for Chagas disease (Reithinger *et al.* 2010), a *T. cruzi* ELISA and a *T. cruzi* IFAT (Pineda *et al.* 2011), and a gold standard based on seropositive reactions for *T. cruzi* by at least two different tests, following WHO recommendation for *T. cruzi* serological diagnosis in humans (Pineda *et al.* 2011).

### Risk factors associated with cutaneous leishmaniasis seropositivity in dogs

To estimate the impact of different risk factors on dog seropositivity patterns we investigated the role of several entomological, ecological and epidemiological variables that were common to dogs belonging to a given household in our study area. We also investigated the joint effect of these household-level covariates with individually collected information from each dog health condition.

For the analysis at the household level we employed maximum likelihood Binomial Generalized Linear Models (Bin-GLMs) (Faraway, 2006). We first identified the best entomological covariate via an Akaike Information Criterion (AIC) comparison (Faraway, 2004) of models, or their simplifications, that considered one of the following entomological variables: (i) total abundance of sand flies collected; (ii) abundance of domiciliary and peridomiciliary sand flies; (iii) total abundance of *Lutzomyia trapidoi* and *Lu. panamensis,* the main dominant vector species in the study area (Calzada *et al.* 2013) and the whole República de Panamá (Christensen *et al.* 1983; Dutari and Loaiza, 2014); (iv) abundance of domiciliary and peridomiciliary *Lu. trapidoi* and *Lu. panamensis*. We needed to perform this preliminary selection of entomological variables given their lack of independence, i.e. some variables are an additive function of the other variables, a fact that can lead to parameter estimation identifiability (Faraway, 2004). We performed model selection via AIC, i.e. considered both model likelihood and parameter number, because models were not always nested (simpler models not always had a subset of variables from a more complex model), therefore not comparable via likelihood ratio tests (Venables and Ripley, 2002).

Following the selection of the best entomological covariate, we proceeded to incorporate all epidemiological and ecological household level variables already described in the Entomological, Epidemiological and Ecological variables at the household level section. We then selected the best model following a procedure of backward elimination from a full model, which considered the best entomological covariate plus all the ecological and epidemiological variables. All variables whose single elimination decreased the simpler model AIC in relation to a model incorporating such covariates were discarded at once (Venables and Ripley, 2002). Nevertheless, at the final stage of model selection we compared nested models (simpler models having a subset of variables from a more complex model) with non-significant factors, employing likelihood ratio tests if the AIC was not minimized by the simplest model (Venables and Ripley, 2002). Given the spatial nature of the households, we performed a Moran I index test on the residuals from the model selected as best, in order to ensure the spatial independence of the residuals, an assumption for the proper use of Bin-GLMs (Venables and Ripley, 2002).

For the individual based risk factor assessment we employed Logistic Generalized Estimating Equations Models (Log-GEEM) (Venables and Ripley, 2002; Faraway, 2006). We employed Log-GEEM given the nature of the data, where dogs belonging to a same household are not independent observations, a fact constraining the use of simpler regression tools (Chaves, 2010). We assumed independence in the correlation structure of the models, given the ability of Log-GEEM to obtain consistent estimates for the fixed effects even when the correlation structure is incorrect (Venables and Ripley, 2002). For the inference we used a sandwich estimator to obtain robust standard errors, since naïve standard errors are appropriate only when the correlation structure is correct (Faraway, 2006). For the identification of significant risk factors for canine cutaneous leishmaniasis, we began our analysis by building a full model that included the best entomological covariate selected for the Bin-GLMs, all the epidemiological and ecological variables collected at the household level and information on each dog health condition (physical condition, cutaneous lesions, de-worming and ectoparasite presence), whether the dog slept inside the house and demography (i.e. sex and age). This model was simplified using a procedure similar to the one employed for the Bin-GLMs, but based on the quasilikelihood information criterion (Pan, 2001), the GEEM analog to AIC.

For the models we employed seropositivity results based on ELISA when fitting both the Bin-GLMs and Log-GEEMs. We did not perform this analysis for the IFAT results alone, given the low sensitivity of this technique (see Results section). Bin-GLMs and Log-GEEMs were fitted with the statistical package R version 2.15.3.

### Force of infection *(*λ*)* and basic reproductive number *(*R_0_*)* estimation

We estimated the force of infection (*λ*) assuming that seroconversion dynamics in susceptible dogs followed an irreversible autocatalytic process (Anderson and May, 1991), which can be described by the following non-linear partial differential equation: (1)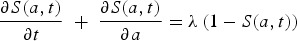 where *S* is the fraction of seropositive dogs in a dog population that is composed by susceptible and seropositive dogs. At any given time, denoted by *t*, equation (1) can be integrated as function of the age, denoted by *a*, and assuming all dogs are born susceptible to become seropositive following the exposure to canine cutaneous leishmaniasis pathogens, we can obtain the following function for the proportion of seropositive dogs (*S*) as function of age (*a*): (2)
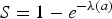


We thus employed our data on seroprevalence (*S*) and age (*a*) to estimate (*λ*) with equation (2). We specifically employed maximum likelihood methods for parameter estimation (Bolker, 2008). Supplement S4 has the R code we employed for the maximum likelihood parameter estimation.

To estimate the basic reproduction number, *R*_0_, we first built a vertical (Southwood, 1978) survival schedule, i.e. a survivorship curve based on the dog population age structure, in order to estimate dog's life expectancy, i.e. the average lifespan (Carey, 2003) of dogs, at Trinidad de Las Minas. Briefly, a vertical survival schedule assumes the age structure of a population to represent the survival schedule of a population at equilibrium and with a pyramidal age structure, i.e. with a larger proportion of younger than older individuals (Southwood, 1978; Krebs, 1998). Because the dog population at Trinidad de Las Minas has a pyramidal structure, and, on average, each household in rural Panama has a couple of dogs (Herrer and Christensen, 1976*b*) it can be argued that dogs are a stationary population fulfilling the assumptions for the sound estimation of a vertical survival schedule, denoted by *l*(*a*), based on the ratios of consecutive age classes: (3)
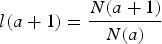


In (3) *l*(*a* + 1) is defined as the probability of surviving from age *a* to *a* + 1 (Carey, 2003). Since individuals at older ages, i.e. 8 or more years, were few, and their abundance did not monotonically decrease, we followed the standard recommendation of smoothing the *l*(*a*) curve (Krebs, 1998). For the smoothing we employed the lowess algorithm (Venables and Ripley, 2002): (4)
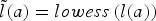


With the smoothed survival schedule we calculated dog's life expectancy, *e*_0_, with the following equation (Southwood, 1978; Krebs, 1998): (5)
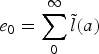


And with *e*_0_, the force of infection (*λ*) and the smoothed survival schedule we estimated *R*_0_ as follows:
(6)
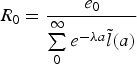
 where (6) makes no specific assumptions about the mortality patterns in the dog population. A detailed derivation of equation (6) and its comparison with expressions that made strong assumptions about population mortality are presented in Supplement S5.

## RESULTS

During our census we counted a total of 52 dogs in the 24 households of our study site, 18 were female and 34 were males. Dogs were present in 22 of the 24 surveyed households, with an average (±s.d.) 2·12 ± 1·42 dog per house. Dog abundance per household ranged from 0 to 6. The average age (±s.d.) of the dogs was 2·07 ± 2·94 years, ranging from less than a month (a puppy still weaning) to 13 years. We took blood samples from all dogs except from the female puppy still weaning that was anaemic and had a very poor body condition score. All subsequent results are based on the 51 animals from which blood samples were taken. We found an ACL seroprevalence of 47% among dogs. About half of the dogs had a poor physical condition (43%). Almost all dogs had ticks attached (94%) and slept outside the houses (92%). Although skin diseases/conditions were observed in 31% of the surveyed dogs, none of them presented clinical lesions or scars suggestive of ACL. Thus, no skin scrapings were analysed for direct parasite detection. Cultures and PCR from blood samples were both negative for *Leishmania* spp. parasites/DNA. A detailed description of the dog population demographic profile, body condition assessment and serodiagnostic test results by age class are presented in Table 1.

**Table 1.** Demography, health condition and cutaneous leishmaniasis seropositivity in a dog population from Trinidad de Las Minas in Panamá

Age – years	Total (females)	Poor physical condition	Ectoparasite presence	Sleeping inside the house	Cutaneous lessons	ELISA+	IFAT+	% Sero-prevalence^a^
Below 1	12 (5)	8	12	1	0	4	1	33 (4)
1–2	16 (6)	6	15	1	7	6	2	38 (6)
3–4	12 (2)	4	10	1	4	7	5	67 (8)
5 or more	11 (4)	4	11	1	4	7	7	73 (8)

a The value inside parenthesis indicates the number of seropositives obtained by at least one method.

The Bayesian analysis employed to evaluate the diagnostic accuracy of the *Leishmania* serological tests, showed the ELISA to outperform IFAT, both in sensitivity and specificity (Table 2). The former has a better ability to capture true seropositive reactions (i.e. sensitivity) close to 80%, while for the latter is 51%. Both ELISA and IFAT tests do not fail to detect pathogen free individuals (i.e. specificity) about 80% of the times. This means that, in general, seropositive patterns detected with ELISA are more likely to be correct (Table S1). A sensitivity analysis for the priors employed in the estimation of sensitivity and specificity of the tests showed the estimates to be robust to assumptions employed in the estimation, nevertheless the sign of the associations between tests outcomes changed (Table S2). However, assumptions about the sign of these associations do not interfere with the proper estimation of sensitivity and specificity, thus further ensuring the soundness of our analysis. Similarly, the multiple Markov chains employed to generate all estimates converged, thus ensuring a valid inferences from our Bayesian Analysis. Further, evidence for the superior quality of the ELISA based diagnosis came from the fact that sera adsorption on *T. cruzi* antigens did not prevent the ELISA *Leishmania* reactions suggesting the lack of positive due to cross-reactions with *T. cruzi,* and the likely co-exposure *L. panamensis* and *T. cruzi* parasites. From 9 positive dogs to *T. cruzi* according to the gold standard of two *T. cruzi* diagnostic tests, 7 dogs were ACL seropositive according to the ELISA test and 9 to the IFAT. The two samples diagnosed as *Leishmania* positive by IFAT and not with ELISA (Table S1) reacted positively with at least two of the Chagas diagnostic tests, thus suggesting a likely cross-reaction. In fact, the Cohen's kappa coefficients of agreement between the *Leishmania* IFAT and the Chagas tests had values above 0·6 (Table S3), which can be considered as a substantial, if above 80% excellent, agreement (Landis and Koch, 1977). In contrast, the agreement between the Chagas tests and the *Leishmania* ELISA was poor (Landis and Koch, 1977), with kappa coefficients below 0·4 in all cases (Table S3). For reference the kappa between the *Leishmania* ELISA and IFAT was 0·415 (Table S3) which is moderate (Landis and Koch, 1977). Thus, given the low sensitivity and specificity of the IFAT, likely derived from the cross-reaction with *T. cruzi*, most subsequent analysis will be based on ELISA results.

**Table 2.** Sensitivity and specificity estimates for canine cutaneous leishmaniasis ELISA and IFAT diagnostic tests. 95% CI indicate the 95% Bayesian credible intervals

Diagnostic test	Parameter	Mean	95% CI
ELISA	Sensitivity	0·79	0·67	0·90
	Specificity	0·84	0·62	0·97
IFAT	Sensitivity	0·51	0·38	0·63
	Specificity	0·77	0·48	0·95

**Fig. 1.**
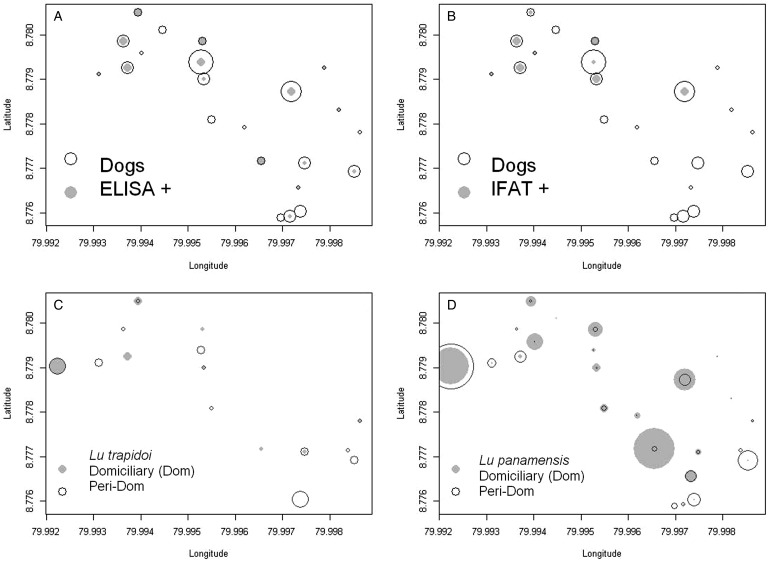
Seroprevalence and dominant vector species abundance. In all panels symbol size is proportional to abundance. (A) ELISA, circles are proportional to the number of dogs (Dogs) and grey dots to the number of ELISA seropositive dogs (ELISA+), symbol size in the inset legend corresponds to two individuals. (B) Indirect Immunofluorescence test, IFAT, circles are proportional to the number of dogs (Dogs) and grey dots to the number of IFAT seropositive dogs (IFAT+), symbol size in the inset legend corresponds to two individuals. (C) *Lutzomyia trapidoi* abundance, for symbol interpretation please refer to the inset legend, where symbol size corresponds to two individuals. (D) *Lu. panamensis* abundance, for symbol interpretation please refer to the inset legend, where symbol size corresponds to four individuals in the domiciliary environment and 20 individuals in the peridomiciliary environment. In the *y-* and *x-*axis 0·001 degree of latitude/longitude are approximately 110 m.

The spatial location of the seropositive dogs according to ELISA (Fig. 1A) and IFAT (Fig. 1B) overlapped with the presence of *Lutzomyia trapidoi* (Fig. 1C) and *Lu. panamensis* (Fig. 1D), especially with the domiciliary abundance of these two species (Fig. 1). Model selection for risk factors at the household level showed that domiciliary *Lu. trapidoi* abundance was the main risk factor associated with ELISA (Table S4) *L*. *panamensis* seropositive diagnostics in a household, with odds for ELISA seropositive diagnosis increasing 2·28 times by each *Lu. trapidoi* sand fly caught by night trap of sand-fly sampling (Table 3). The Moran's I test supports the lack of spatial autocorrelation in residuals from the binomial generalized linear models we fitted in Table 3, a fact that, in addition to the confirmation of generalized linear model assumptions about the error, suggests our inferences are sound. The analysis from data gathered for the individual dogs only included sex, age, skin lesion presence, deworming and physical condition. We excluded tick presence and sleeping outside the house given their low variability. Model selection showed that for ELISA (Table S5) only domiciliary *Lu. trapidoi* abundance by trap-night and age were significant risk factors. In the case of ELISA the odds of seropositive reaction in a given dog increased 3·39 times and 1·35 times, respectively, by each sand fly caught inside the house where a dog belonged and dog age in years (Table 4).

**Table 3.** Parameter estimates for the best binomial generalized linear models explaining the odds ratio of cutaneous leishmaniasis ELISA seropositive reactions in dogs at the household level. 95% CI indicate the 95% maximum likelihood confidence intervals for the estimated odds

Parameter	Odds ratio (95% CI)	Estimate (±s.e.)	*P*
Intercept	1	–	–
*Lutzomia trapidoi* abundance inside the houses	2·28 (1·07–7·13)	0·948 (±0·473)	0·033*
Moran's I test for spatial autocorrelation in the residuals	–	−0·085	0·568

* Statistically significant (*P* < 0·05).

**Table 4.** Parameter estimates for the best logistic generalized estimating equation models explaining cutaneous leishmaniasis seropositivity by ELISA in dogs. Houses were considered as the clustering factor in the analysis

Parameter	Odds ratio	Estimate (±sandwich s.e.)	*Z*
Intercept	1	–	–
*Lutzomia trapidoi* abundance inside the houses	3·39	1·22 (±0·39)	3·122*
Dog age	1·35	0·30 (±0·09)	3·395*

* Statistically significant (*P* < 0·05).

**Fig. 2.**
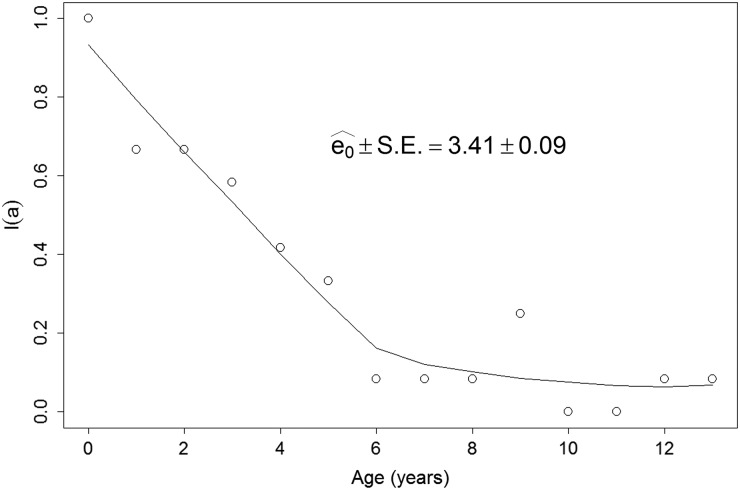
Age specific survival (*l*(*a*)) schedule from a vertical life table. Open circles are the raw estimates from the data. The solid line represents a lowess smoothed survival schedule. Life expectancy (*e*_0_) was estimated with the lowess smoothed survival *l*(*a*) curve and equation (5).

**Fig. 3.**
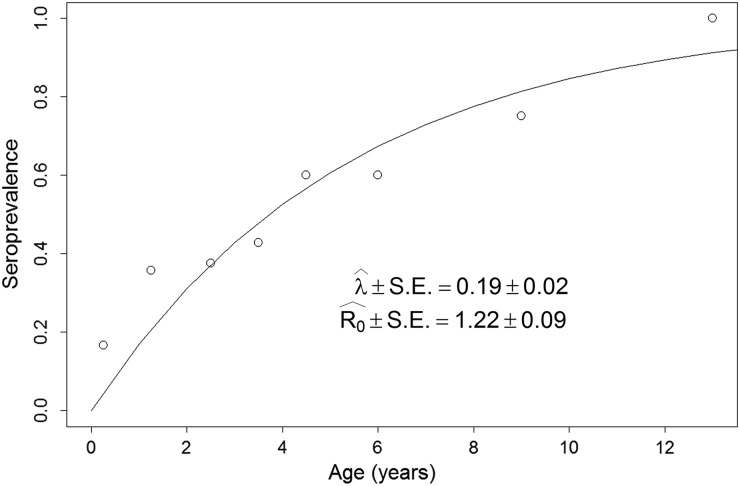
Force of infection (*λ*) and basic reproduction number (*R*_0_) estimates. Estimates are based on the age specific seroprevalence from ELISA. *λ* was estimated via the maximum likelihood fitting of equation (2) to the seroprevalence data (open circles). For a full description of the maximum likelihood procedure see Supplement S4. *R*_0_ was estimated with equation (6).

The vertical life table we built based on the dog population age profile, estimated dog age expectation at birth (*e*_0_) to be close to three and a half years (Fig. 2). Figure 3 shows the estimated age specific seroprevalence curves, force of infection (*λ*) and basic reproductive number (*R*_0_) with ELISA. *R*_0_ was significantly larger than 1, indicating that the disease was endemically established in dogs when our survey was done and that, on average, new infections will keep appearing in a fully susceptible dog population, *ceteris paribus* (i.e. everything else being equal). Finally, *R*_0_ estimates (Fig. 3) by equation (6) highlight how estimates based on the ‘extreme’ assumptions of low mortality to the age expectation at birth or constant age independent mortality are likely biased (Table S6), making preferable the use of the population survival schedule when available.

## DISCUSSION

As a zoonosis ACL is particularly interesting given the diversity of hosts that can be infected by NWC *Leishmania* spp. parasites (Garnham, 1965; Ashford, 1997). Not all host species are reservoirs of infection, some species are incidental hosts, i.e. dead-end hosts from which the parasites would unlikely infect vectors, while, by contrast, other species are true reservoirs, with a dynamic feedback in the transmission between sand fly vectors and the vertebrate hosts (Chaves and Hernandez, 2004; Chaves *et al.* 2007, 2008*b*). The incrimination of reservoir hosts in ACL transmission critically depends on high quality diagnostics, which over recent years have significantly evolved. Nevertheless, the absence of a gold standard has remained a major challenge (Reithinger *et al.* 2000; Reithinger and Davies, 2002), which nowadays can be partially circumvented due to the development of Bayesian statistical tools, that allow the estimation of sensitivity and specificity in the absence of a gold standard (Branscum *et al.* 2005). In our study, we benefited from these ‘new’ Bayesian techniques and were able to confirm that a *L. panamensis* ELISA was a diagnostic tool able to outperform the commonly employed IFAT, an observation in accordance with several previous studies (Reithinger and Davies, 1999; Castro *et al.* 2007). Nevertheless, we consider extremely desirable the development of even more sensitive and specific diagnostic tests. For example, rapid diagnostic tests like the rK39 RDT recently developed for canine visceral leishmaniasis (Quinnell *et al.* 2013) can significantly improve studies evaluating the reservoir role of dogs across the distinct ACL eco-epidemiological settings. However, the potential of recombinant antigens and/or rapid tests for *Leishmania Viannia* spp. serological diagnosis has not been validated yet.

The performance (sensitivity and specificity) of serological diagnostics methods are influenced significantly by the characteristics of the antigen used. In this study we used *L. panamensis* promastigotes (IFAT) or their soluble crude extracts (ELISA) following procedures previously described and recommended by the World Organization for Animal Health. Our analysis showed that IFAT was more likely to cross-react with *T. cruzi*, which is a problem for proper diagnosis in areas where ACL and Chagas disease co-occur (Padilla *et al.* 2002), further reinforcing the use of ELISA-like and other sensitive and specific diagnostics. New advances for the identification of *Leishmania* species with polymorphism specific PCR (Marco *et al.* 2012) can also greatly improve the understanding of circulating parasite species in endemic areas. In that sense, our study was limited given that we can only assume that the circulating species was *L. panamensis*, the species we were able to identify in biopsies and skin scrapings from humans with active skin lesions living in the studied area (Saldaña *et al.* 2013).

Results about risk factors for the exposure to NWC *Leishmania* spp. were within what could be expected. Seropositive reactions, indicative of exposure to NWC *Leishmania* spp. parasites, were more likely to occur in houses with *Lu. trapidoi*, and further increased with the abundance of this dominant vector species, the main ACL vector in the Republic of Panamá (Christensen *et al.* 1983; Dutari and Loaiza, 2014) and one of the dominant vector species at our study site (Calzada *et al.* 2013; Chaves *et al.* 2013; Saldaña *et al.* 2013). The skin lesions we observed in dogs were not typical of ACL. This might reflect that in tropical regions, like our study site, skin diseases in dogs due to ectoparasites, bacteria and fungus are very frequent and may result in similar skin lesions, complicating the clinical diagnosis of ACL (Fung *et al.* 2014).

Also, the exposure risk increased with domiciliary sand fly abundance, probably reflecting the fact that dogs sleep just outside the households, a place where sand fly density is likely more closely associated with the intra-domiciliary sand fly abundance than with the peridomiciliary abundance sampled by a trap within 50 m from the household, especially in light of the poor dispersal ability of sand flies, which occurs mainly within 20 m (Alexander, 1987; Morrison *et al.* 1993). Interestingly, previous studies in Panama observed that *Lu. trapidoi* was attracted to dogs, humans, but also to wildlife species that could act as NWC *Leishmania* spp. reservoirs (Christensen and Herrer, 1973; Herrer and Christensen, 1976*a*, *b*). In our study site, the risk for clinical human ACL increased with the abundance of *Lu. gomezi* and *Lu. trapidoi* (Saldaña *et al.* 2013), both NWC *Leishmania* spp. competent sand fly vectors (Dutari and Loaiza, 2014). These observations deserve to be placed in light of our previous findings, since trends of sand fly infestation at our study site are closely related with housing quality. Destitute houses were more likely to be re-infested by sand flies after vector control (Chaves *et al.* 2013), thus highlighting that domestic dog exposure to NWC *Leishmania* spp. parasites might be linked with the poverty context of ACL transmission. Exposure also increased with age, probably reflecting the accumulation of infective bites through the lifespan of a dog in an endemic area (Quinnell *et al.* 1997), a pattern observed in endemic areas for other vector-borne diseases (Anderson and May, 1991).

Our model to study age specific canine ACL seroprevalence patterns had two major differences with a previous study (Reithinger *et al.* 2003*a*). To the best of our knowledge, that study has been the only attempt to model and estimate the force of infection and *R*_0_ of canine ACL in dogs. First, we did not consider the possibility of recovery in dogs, i.e. that seropositive dogs could become seronegative, which is a limitation of cross-sectional studies like ours that cannot measure seronegative conversions. Nevertheless, Reithinger *et al.* (2003*a*) present parameters that were not significantly different from 0, suggesting that likelihood of recovery is very small, and that temporal variability in the samples was not properly accounted when assigning ACL seropositivity to dogs. Observations by Marco *et al.* (2001) indeed suggest that negative seroconversion is highly unlikely, and Aguilar *et al.* (1989) found that some temporal variability might exist leading to inconsistent serodiagnosis in cohort studies, suggesting that ACL exposure leads to long-lasting seroconversion. In addition, this might happen when transmission is endemic and exposure is sustained. In that sense we want to highlight that we used a cutting edge method to assign seropositive status to our ELISA samples (Bretscher *et al.* 2013) and that, for the purpose of an abstraction (Levins, 1968, 2006), not considering dog recovery, given that dog mortality is likely a more important process regulating transmission in dogs, does not invalidate our model. Second, we did not consider dog mortality as a constant, nor did we use models assuming that mortality was constant across ages (Quinnell *et al.* 1997; Reithinger *et al.* 2003*a*). The landmark work by Anderson and May (1991), derived results assuming a constant mortality given the elegance of closed form solutions that can be expressed with simple formulas. Nevertheless, in no instance Anderson and May (1991) reified this to be the case in nature. Our data clearly showed that estimates using the survival schedule can produce estimates (Fig. 3) contained between the extreme cases derived by Anderson and May (1991) (Table S6). Thus, our estimates are likely more accurate and can more realistically account for the non-constant nature of mortality observed across organisms (Carey, 2001, 2003). Similarly, although there were no significant differences between the *R*_0_ estimates based on ELISA and ELISA or IFAT diagnostics, the poor specificity, and potential cross-reactivity with *T. cruzi*, of the IFAT highlights how the pooling of results by different diagnostic tests (Reithinger *et al.* 2003*a*) may not be warranted, given underlying differences in data quality (Boggild *et al.* 2010).

Age specific seroprevalence patterns suggest that canine ACL was endemically established at Trinidad de Las Minas when our cross-sectional study took place. *R*_0_, the basic reproduction number, was significantly above 1, meaning that exposure of dogs to NWC *Leishmania* spp. parasites was endemically established (Chaves and Hernandez, 2004). Here, we want to stress that endemic establishment does not imply that dogs are reservoirs, since as long as the disease is endemically established in the true reservoirs (Chaves *et al.* 2008*b*), cutaneous leishmaniasis can be expected to be established in any incidental host that gets into frequent contact with infected sand flies (Chaves *et al.* 2007). Here, it is also important to bring results from previous studies in Panamá, where vectors associated with human transmission do not often feed in dogs (Telford Jr *et al.* 1972; Herrer and Christensen, 1976*a*, *b*; Christensen and Herrer, 1980), unlike the two toed sloth, which showed a dynamical feedback of transmission with sand fly vectors, with a high success probability of vector infection (Thatcher and Hertig, 1966; Christensen and Herrer, 1972, 1979; Herrer and Christensen, 1980) significantly higher than what has been observed in dogs (Hernández *et al.* 2006; Travi *et al.* 2006) or humans (Rojas and Scorza, 1990). In fact, we observed two toed sloths in our study area, from which we were able to isolate *L. panamensis* parasites (González *et al.* 2015), a competent reservoir from which *L. panamensis* has been previously isolated (Herrer and Christensen, 1980). In summary, the presence of alternative likely reservoirs hosts, the relative low attraction of sand fly vectors to dogs when compared with sloths, the low probability of sand fly infection on dogs, as well as the low haematogeneicity of NWC *Leishmania* spp. parasites (Pirmez *et al.* 1988; Reithinger *et al.* 2000, 2002), low parasite density that has been detected in blood (Padilla *et al.* 2002) and given that most ACL in Panamá is due to infections with *L. panamensis* (Chaves *et al.* 2014) we think that dogs are not a significant reservoir in the *L. panamensis* ACL epidemiology in our study site, which is likely illustrative of ACL epidemiology in the whole Republic of Panamá.

## Supplementary material

For supplementary material accompanying this paper visit http://dx.doi.org/10.1017/S0031182015000475. click here to view supplementary material
