# Supplementary material for: Cutaneous Leishmaniasis in dogs: is high seroprevalence indicative of a reservoir role?
Source: Parasitology. 2015 May 20;142(9):1202–14. doi: 10.1017/S0031182015000475 (PMC4531497; doi:10.1017/S0031182015000475)
Supplement: Supplementary file 1 [file S0031182015000475sup001.zip › Table S6.pdf]

Table S6 R_0 estimates assuming an insignificant or a constant mortality

Mortality Assumption	Diagnostic	R_0	S.E.
Insignificant mortality up to the life expectancy age (equation S5.10 in supplement S5)	ELISA	0.64	0.085
Constant (equation S5.14 in supplement S5)	ELISA	1.64	0.085
