# Supplementary material for: Cutaneous Leishmaniasis in dogs: is high seroprevalence indicative of a reservoir role?
Source: Parasitology. 2015 May 20;142(9):1202–14. doi: 10.1017/S0031182015000475 (PMC4531497; doi:10.1017/S0031182015000475)
Supplement: Supplementary file 1 [file S0031182015000475sup001.zip › Table S3 Kappa coefficient of agreement between the serological diagnostic tests for Trypanosoma cruzi.pdf]

Table S3 Kappa coefficient of agreement between the serological diagnostic tests for *Trypanosoma cruzi* (Chagas) and *Leishmania (Viannia)* spp. exposure

Diagnostic test	Kappa	Z	P
Leishmania ELISA – Leishmania IFAT	0.415	3. 14	0.00167 *
Leishmania ELISA – Chagas	0.268	2.31	<0.0208*
Leishmania ELISA – Chagas IFAT	0.315	2.63	<0.00866*
Leishmania ELISA – Chagas ELISA	0.230	1.91	0.0550
Leishmania ELISA – Chagas Rapid Test	0.221	1.98	0.0475*
Leishmania IFAT – Chagas	0.723	5.37	<0.00001*
Leishmania IFAT – Chagas IFAT	0.784	5.73	<0.00001*
Leishmania IFAT – Chagas ELISA	0.568	4.15	<0.00003
Leishmania IFAT – Chagas Rapid Test	0.659	5.01	<0.00001*
Chagas Rapid Test – Chagas IFAT – Chagas ELISA	0.781	9.67	<0.00001*

*Statistically significant (P<0.05)
