# Supplementary material for: Cutaneous Leishmaniasis in dogs: is high seroprevalence indicative of a reservoir role?
Source: Parasitology. 2015 May 20;142(9):1202–14. doi: 10.1017/S0031182015000475 (PMC4531497; doi:10.1017/S0031182015000475)
Supplement: Supplementary file 1 [file S0031182015000475sup001.zip › Table S2 Diagnostic test sensitivity and specificity parameter estimate sensitivity to changes in the prior distribution.pdf]

Table S2 Diagnostic test sensitivity and specificity parameter estimate sensitivity to changes in the prior distribution. In the table Prior indicates the parameters used for the Beta prior distribution, Parameter the name of the parameter (rhoD is the correlation between positive results and rhoDc between negative results), Test the name of the diagnostic test and 95 % CI, the 95 % Bayesian credible intervals of the estimates. Further details about the sensitivity analysis are presented in Supplement S1.

Prior	Parameter	Test	95 % CI	
Beta(α=15.034,β=2.559)[+]	Sensitivity	ELISA	0.67	0.90
Beta(α=9.628,β=3.876)[+]		IFAT	0.38	0.64
Beta(α=15.034,β=2.559)[+]	Specificity	ELISA	0.62	0.97
Beta(α=10.034,β=2.559)[+]		IFAT	0.48	0.95
----	rhoD	---	-0.59	-0.13
----	rhoDc	---	-0.39	0.77
Beta(α=15.034,β=2.559)	Sensitivity	ELISA	0.69	0.95
Beta(α=9.628,β=3.876)		IFAT	0.36	0.63
Beta(α=15.034,β=2.559)	Specificity	ELISA	0.60	0.97
Beta(α=5.034,β=2.559)		IFAT	0.24	0.90
----	rhoD	---	-0.57	0.04
----	rhoDc	---	-0.71	0.65
Beta(α=15.034,β=2.559)	Sensitivity	ELISA	0.68	0.88
Beta(α=6.628,β=3.876)		IFAT	0.36	0.61
Beta(α=15.034,β=2.559)	Specificity	ELISA	0.63	0.97
Beta(α=10.034,β=2.559)		IFAT	0.62	0.97
----	rhoD	---	-0.60	-0.18
----	rhoDc	---	-0.25	0.80
Beta(α=15.034,β=2.559)	Sensitivity	ELISA	0.69	0.89
Beta(α=3.628,β=3.876)		IFAT	0.33	0.59
Beta(α=15.034,β=2.559)	Specificity	ELISA	0.64	0.97
Beta(α=10.034,β=2.559)		IFAT	0.61	0.97
----	rhoD	---	-0.61	-0.18
----	rhoDc	---	-0.24	0.80
Beta(α=15.034,β=2.559)	Sensitivity	ELISA	0.67	0.89
Beta(α=9.628,β=3.876)		IFAT	0.39	0.67
Beta(α=10.034,β=2.559)	Specificity	ELISA	0.43	0.96
Beta(α=10.034,β=2.559)		IFAT	0.61	0.97
Beta(α=15.034,β=2.559)	Sensitivity	ELISA	0.66	0.95
Beta(α=9.628,β=3.876)		IFAT	0.41	0.87
Beta(α=5.034,β=2.559)	Specificity	ELISA	0.20	0.88
Beta(α=10.034,β=2.559)		IFAT	0.58	0.96
----	rhoD	---	-0.56	0.49
----	rhoDc	---	-0.90	0.59

[+]Results Presented in Table 1 of the main article
