# Supplementary material for: Cutaneous Leishmaniasis in dogs: is high seroprevalence indicative of a reservoir role?
Source: Parasitology. 2015 May 20;142(9):1202–14. doi: 10.1017/S0031182015000475 (PMC4531497; doi:10.1017/S0031182015000475)
Supplement: Supplementary file 1 [file S0031182015000475sup001.zip › Table S1 Cross.pdf]

Table S1 Cross-classified canine cutaneous leishmaniasis diagnostic test results from ELISA and IFAT.

Diagnostic Test		ELISA +	ELISA -
IFAT	+	13	2
	-	11	25
