# Supplementary material for: Cutaneous Leishmaniasis in dogs: is high seroprevalence indicative of a reservoir role?
Source: Parasitology. 2015 May 20;142(9):1202–14. doi: 10.1017/S0031182015000475 (PMC4531497; doi:10.1017/S0031182015000475)
Supplement: Supplementary file 1 [file S0031182015000475sup001.zip › Supplement S5 Basic Reproduction number.pdf]

Supplement S5 Basic Reproduction number (R_0) estimation when mortality is neither constant nor negligible and the force of infection (λ) is age independent and assumed to be at equilibrium

Anderson and May (1991) present that in a homogeneously mixed host population, under the assumption of "weak homogenous mixing", i.e., that new infections appear at rate proportional to the number of susceptible hosts, R_0 is inversely related to the fraction of susceptible hosts ($S*$) at equilibrium:

$$R_0 = \frac{1}{S^*} \tag{S5.1}$$

The fraction of susceptible hosts ($S*$) at equilibrium can be decomposed as a function of the total number of susceptible hosts ($\overline{X}$) divided the total number of hosts ($\overline{N}$), over all ages (a), as follows:

$$S^* = \frac{\overline{X}}{\overline{N}} = \frac{\int_0^\infty X(a)da}{\int_0^\infty N(a)da} \tag{S5.2}$$

Where $\overline{N}$ is an age structured population that can be decomposed as follows:

$$\overline{N} = \sum_0^\infty N(0)l(a) \tag{S5.3}$$

Which can be further simplified to:

$$\overline{N} = N(0)\sum_0^\infty l(a) \tag{S5.4}$$

Similarly, $\overline{X}$ is the age structured population of infected individuals that can be decomposed as follows:

$$\overline{X} = N(0)\sum_0^\infty e^{-\lambda a} l(a) \tag{S5.5}$$

Substituting (S5.4) and (S5.5) in (S5.2) and (S5.1) we have:

$$R_0 = \frac{e_0}{\sum_0^\infty e^{-\lambda a} l(a)} \tag{S5.6}$$

Where the numerator is the life expectancy in a population (e_0), i.e., the expected time units a newborn will survive until his/her death (Carey, 2001; Carey, 2003), which is defined by the following equation:

$$e_0 = \sum_0^\infty l(a) \tag{S5.7}$$

Equation S5.6 can be approximated under two limit considerations(Anderson & May, 1991):

(1) When mortality is negligible up to the life expectancy age (*e_0*, *$l(a< e_0)$*=1)*,* after which survival becomes negligible(i.e., *$l(a> e_0)$*=0):

$$R_0 = \frac{e_0}{\sum_0^w e^{-\lambda a} l(a)} = \frac{e_0}{\frac{1}{\lambda}\left(1-e^{-\lambda e_0}\right)} \quad \text{(S5.8)}$$

Where S5.8 can be fairly well approximated by:

$$R_0 = \lambda e_0 \quad \text{(S5.9)}$$

Or can be re-expressed as the ratio between the age expected age for the first infection (*A*):

$$R_0 = \frac{e_0}{A} \quad \text{(S5.10)}$$

Since under the static assumptions(Anderson & May, 1991):

$$A = \frac{1}{\lambda} \quad \text{(S5.11)}$$

(2) When mortality is constant through all ages (*$l(a)=-exp(\mu a)$*)*:*

$$R_0 = \frac{e_0}{\sum_0^\infty e^{-\lambda a} e^{-\mu a}} = e_0(\lambda + \mu) \quad \text{(S5.12)}$$

Since the life expectancy (e_0) is also the inverse of (μ), the average mortality rate (Carey, 2003):

$$e_0 = \frac{1}{\mu} \quad \text{(S5.13)}$$

And replacing S5.12 in S5.11 we have:

$$R_0 = 1 + \frac{e_0}{A} \quad \text{(S5.14)}$$

An equation that has been used when estimating R_0 for leishmaniasis transmission in dogs (Quinnell *et al.*, 1997; Reithinger *et al.*, 2003). Nevertheless, none of those approximations are likely good to estimate R_0 in populations where mortality accelerates with age, a pattern observed in nature (Carey, 2003) or when mortality is, more generally, not constant. In those circumstances, the best estimate would probably to use equation (S5.6) which can estimate R_0 in absence of the biases imposed by assumptions of equations (S5.10) and (S5.14). Nevertheless, it is important to highlight that equation

S5.6 assumes: (i) homogenous pathogen exposure, (ii) no significant delay between infection and seropositivity, (iii) the force of infection is constant and age independent, and (iv) infection and demographic patterns in the host population are at equilibrium(Anderson & May, 1991).

References

Anderson, R. M. and May, R. M. (1991). *Infectious diseases of humans: dynamics and control,* OXford University Press, Oxford.

Carey, J. R. (2001). Insect biodemography. *Annual Review of Entomology,* **46**, 79-110.

Carey, J. R. (2003). *Longevity: the biology and Demography of life span,* Princeton University Press, Princeton, NJ, USA.

Quinnell, R. J., Courtenay, O., Garcez, L. and Dye, C. (1997). The epidemiology of canine leishmaniasis: transmission rates estimated from a cohort study in Amazonian Brazil. *Parasitology,* **115**, 143-156. doi: doi:10.1017/S0031182097001200.

Reithinger, R., Espinoza, J. C. and Davies, C. R. (2003). The transmission dynamics of canine american cutaneous Leishmaniasis in Huánuco, Perú. *The American Journal of Tropical Medicine and Hygiene,* **69**, 473-480.
