# Supplementary material for: Cutaneous Leishmaniasis in dogs: is high seroprevalence indicative of a reservoir role?
Source: Parasitology. 2015 May 20;142(9):1202–14. doi: 10.1017/S0031182015000475 (PMC4531497; doi:10.1017/S0031182015000475)
Supplement: Supplementary file 1 [file S0031182015000475sup001.zip › Supplement S4 R code for the maximum likelihood estimation of the force of infection.pdf]

Supplement S4 R code for the maximum likelihood estimation of the force of infection (λ) from an age-specific seroprevalence curve

```
library(bbmle)

### Model fitting requires the use of the bbmle library

sero<-function(lambda,sd){
-sum(dnorm(seroprevalence,mean=(1-exp(lambda*age)),sd=sd,log=T))
}

### sero is the maximum likelihood function to be optimized. We assumed that
### seroprevalence was normally distributed Its parameters are lambda which
### corresponds to λ in equation (2) and sd, which is the standard deviation
### of the error.
### For the maximum likelihood estimation we need a vector with age specific
### seroprevalence and age to define the relations presented in equation 2.
### the sum corresponds the log-likelihood expression for the model, i.e.,
### $L(S(a)|\lambda, var) = \sum_{a=0}^{\omega} \kappa - \frac{(\omega+1)\ln(sd)}{2} - \frac{1}{2sd^2}\sum_{a=0}^{w}\left(S(a) - \left(1 - e^{-\lambda a}\right)\right)^2$, where κ is a constant
### from the normal distribution, ω is the maximum age in the dog population
### and sd² is the variance of the error.

modelfit=mle2(sero,start=list(lambda=-.16,sd=0.14))

### mle2 is a command to fit the maximum likelihood model, with initial
### guesses for the parameters defined in “start=list(…)”

summary(modelfit)

### summary is a command that summarizes the output from the maximum
### likelihood estimation


confint(modelfit)

### confint is a command that estimates the 95% confidence intervals for the
### parameters in the maximum likelihood function via maximum likelihood
### profiling.
```
