# Supplementary material for: Cutaneous Leishmaniasis in dogs: is high seroprevalence indicative of a reservoir role?
Source: Parasitology. 2015 May 20;142(9):1202–14. doi: 10.1017/S0031182015000475 (PMC4531497; doi:10.1017/S0031182015000475)
Supplement: Supplementary file 1 [file S0031182015000475sup001.zip › Supplement S2 The Dendukuri and Joseph method to estimate sensitivity and specificity of two diagnostic tests in one Population.pdf]

Supplement S2 The Dendukuri and Joseph method to estimate sensitivity and specificity of two diagnostic tests in one Population

The Dendukuri and Joseph method is based on the Hui and Walter (HW) equations (Hui & Walter, 1980). The HW method allows the estimation of sensitivity and specificity of two diagnostic tests in two populations using the number of double positives (i.e., positively diagnosed individuals by the two tests), double negatives, and those positive by only one of the diagnostic tests (presented in Table S1 for our study data). Briefly, the Dendukuri and Joseph (Dendukuri & Joseph, 2001) method modifies the HW equations to obtain estimates in the presence of non-independent diagnostics, i.e., diagnostics whose sensitivity and/or specificity are likely correlated. Given lack of degrees of freedom for maximum likelihood parameter estimation when the two tests are performed on the same population (seven parameters on four observations), the subsequent non-identifiability of the parameters, and the bias of the HW maximum likelihood estimator when diagnostic tests are correlated (Branscum *et al.*, 2005; Dendukuri & Joseph, 2001; Johnson *et al.*, 2001), inferences for the Dendukuri and Joseph method require a Bayesian framework for parameter inference.

Dendukuri and Joseph (Dendukuri & Joseph, 2001), as well as Branscum et al (Branscum *et al.*, 2005), also recognized the likely large influence of the informative priors required for the estimation of identifiable parameters in their modified HW equations. To better understand the role of the prior distributions on parameter estimates, Dendukuri and Joseph (Dendukuri & Joseph, 2001) and Branscum et al (Branscum *et al.*, 2005) recommend to perform a sensitivity test increasing the range for quantile distribution in the priors assumed for the specificity and sensitivity. To achieve such a goal we decreased the value of the α parameter in the beta distribution defining the prior distribution for the specificity of ELISA and IFAT, and the sensitivity of IFAT. Results are presented in Table S2.

References

Branscum, A. J., Gardner, I. A. and Johnson, W. O. (2005). Estimation of diagnostic-test sensitivity and specificity through Bayesian modeling. *Preventive Veterinary Medicine,* **68**, 145-163. doi: http://dx.doi.org/10.1016/j.prevetmed.2004.12.005.

Dendukuri, N. and Joseph, L. (2001). Bayesian approaches to modeling the conditional dependence between multiple diagnostic tests. *Biometrics,* **57**, 158-167.

Hui, S. L. and Walter, S. D. (1980). Estimating the error rates of diagnostic tests. *Biometrics,* **36**, 167-171.

Johnson, W. O., Gastwirth, J. L. and Pearson, L. M. (2001). Screening without a "gold standard": The Hui-Walter paradigm revisited. *American Journal of Epidemiology,* **153**, 921-924.
