# Supplementary material for: Cutaneous Leishmaniasis in dogs: is high seroprevalence indicative of a reservoir role?
Source: Parasitology. 2015 May 20;142(9):1202–14. doi: 10.1017/S0031182015000475 (PMC4531497; doi:10.1017/S0031182015000475)
Supplement: Supplementary file 1 [file S0031182015000475sup001.zip › Supplement S1 Seropositivity thresholds from mixture models.pdf]

Supplement S1 Seropositivity thresholds from mixture models

Standard methodologies for seropositivity were usually based on control groups, from which a threshold for seropositivity was obtained by adding three standard deviations to their mean antibody titre (Dye *et al.*, 1993). Nevertheless, this method was prone to be biased and misclassify seropositive and seronegative individuals because it is not robust to small changes in antibody titres that can emerge from seasonality and/or small variations in laboratory assay performance (Bretscher *et al.*, 2013). To overcome the likelihood of misclassification, a novel method based on a finite mixture model has been proposed to determine a more sensitive threshold for seropositivity based on the distribution of titres, measured via optical density in a spectrophotometer, in a population exposed to a pathogen (Bretscher *et al.*, 2013; Stewart *et al.*, 2009). Basically, the finite mixture model assumes the statistical distribution ($f(x)$) of antibody titres, or optical densities, (x) in an exposed population to be a mixture of the distribution of titres from positive ($P(x)$) and negative individuals ($N(x)$) according to the following equation (Bretscher *et al.*, 2013; Stewart *et al.*, 2009):

$$f(x) = \pi(P(x)) + (1-\pi)(N(x)) \qquad \text{(S1.1)}$$

Where π is the proportion of titres belonging to each category. Maximum likelihood estimates for the means and standard deviations of P and N distributions can be estimated and a threshold for seronegativity can be estimated as the mean of the distribution with the smallest mean (i.e., that of the negative individuals) plus three standard deviations (Stewart *et al.*, 2009). Parameters for the finite mixture model were estimated with the command normalmixEM() of the library mixtools in the statistical package R version 3.0.1.

References

Bretscher, M., Supargiyono, S., Wijayanti, M., Nugraheni, D., Widyastuti, A., Lobo, N., Hawley, W., Cook, J. and Drakeley, C. (2013). Measurement of *Plasmodium falciparum* transmission intensity using serological cohort data from Indonesian schoolchildren. *Malaria Journal,* **12**, 21.

Dye, C., Vidor, E. and Dereure, J. (1993). Serological diagnosis of leishmaniasis: on detecting infection as well as disease. *Epidemiology & Infection,* **110**, 647-656. doi: doi:10.1017/S0950268800051074.

Stewart, L., Gosling, R., Griffin, J., Gesase, S., Campo, J., Hashim, R., Masika, P., Mosha, J., Bousema, T., Shekalaghe, S., Cook, J., Corran, P., Ghani, A., Riley, E. M. and Drakeley, C. (2009). Rapid Assessment of Malaria Transmission Using Age-Specific Sero-Conversion Rates. *PLoS ONE,* **4**, e6083. doi: 10.1371/journal.pone.0006083.
