# Supplementary figures and images for: Cutaneous Leishmaniasis in dogs: is high seroprevalence indicative of a reservoir role?
Source: Parasitology. 2015 May 20;142(9):1202–14. doi: 10.1017/S0031182015000475 (PMC4531497; doi:10.1017/S0031182015000475)

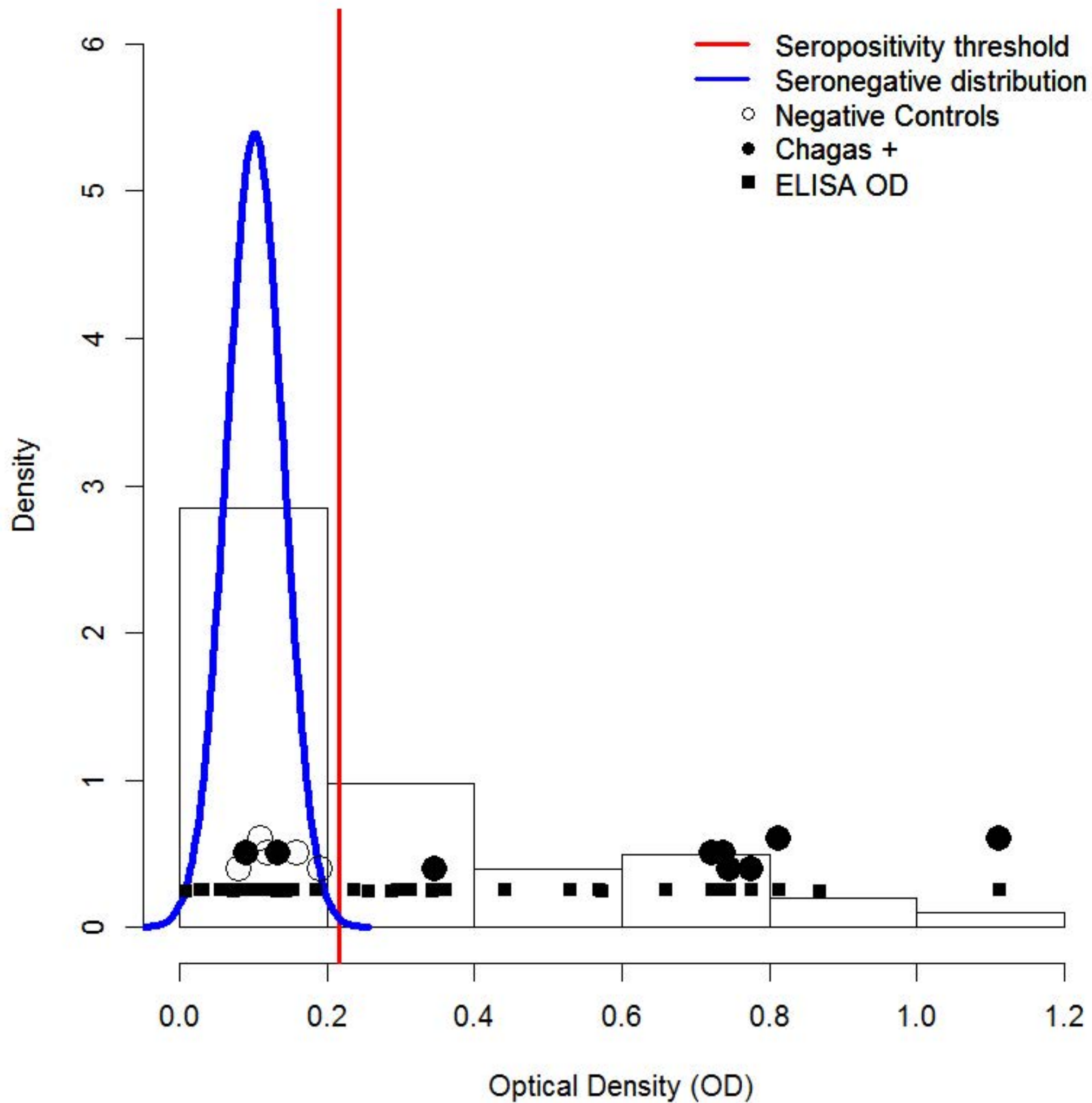

Supplement: Supplementary file 1 [file S0031182015000475sup001.zip › PAR1500047 NEW SUPP MAT file for FigS1.pdf]
